# The Benefits of the Positive Parenting Program as Early Intervention for Mothers of Children Aged 1–3 Years Who May Have Neurodevelopmental Disorders

**DOI:** 10.3390/children13040469

**Published:** 2026-03-28

**Authors:** Hiromi Masuda, Kazuaki Tanabe, Yukari Nakano

**Affiliations:** 1 Department of Perioperative and Critical Care Management, Graduate School of Biomedical and Health Sciences, Hiroshima University, Hiroshima 734-8553, Japan; ktanabe2@hiroshima-u.ac.jp (K.T.); nakano@catherine.ac.jp (Y.N.); 2 Master’s Course in Nursing, Graduate School of Nursing, St. Catherine University, Matsuyama 790-0022, Japan; 3 Department of Childcare, Junior College, St. Catherine University, Matsuyama 799-2496, Japan

**Keywords:** neurodevelopmental disorders, parenting, infants and toddlers, community parenting support, early intervention, mental health, Triple P (positive parenting program)

## Abstract

**Highlights:**

**What are the main findings?**

- The Group Triple P (the Positive Parenting Program) program increased mothers’ recognition of positive behaviors in young children who may have neurodevelopmental disorders and reduced dysfunctional parenting practices.
- GTP restored mothers’ confidence and fulfilling sense in parenting, and decreased maternal depression and stress. Perceived access to parenting support also improved, contributing to better parenting adaptation.

**What are the implications of the main findings?**

- As a community-based early intervention for families with infants and toddlers who may have neurodevelopmental disorders, GTP enhanced mothers’ confidence and sense of fulfillment in parenting, along with willingness to seek support, thereby supporting parental mental health and fostering more resilient parenting environments.
- GTP is a scalable, community-based group intervention program designed for families awaiting a definitive diagnosis; as a population-based selective approach, it has the potential to be proposed as a form of early intervention.

**Abstract:**

**Background/Objectives**: The Positive Parenting Program (Triple P) is an evidence-based intervention for families including young children with neurodevelopmental disorders. Because establishing a definitive diagnosis in early childhood takes time, especially for 1–3-year-olds who have only recently shown early signs, parents often experience substantial stress. This study examines the effectiveness of the Group Triple P (GTP) program for mothers of 1–3-year-old children without a confirmed diagnosis who spent time at a community parenting support center. **Methods**: The participants were 41 mothers of children aged 1–3 who did not confirmed neurodevelopmental diagnosis but showed behavioral, emotional, or developmental concerns, or whose mothers reported parenting difficulties. To reflect real community practices, a non-randomized pre–post test design without a control group was adopted. Assessments were conducted at baseline, postintervention, and at a 12 weeks follow-up using the Strengths and Difficulties Questionnaire, Parenting Scale, Parenting Experience Scale, and Depression Anxiety Stress Scale. **Results**: GTP improved the mothers’ assessments of positive behaviors in children who may have neurodevelopmental disorders, with a medium effect size. GTP reduced dysfunctional parenting styles in mothers with a large effect size. It also enhanced mothers’ confidence and fulfilling sense in parenting, and decreased depression and stress with a medium effect size. Perceived access to parenting support improved, contributing to better parenting adaptation with a medium effect size. **Conclusions**: GTP may serve as an early public health intervention for mothers of young children who may have neurodevelopmental disorders by supporting maternal mental health and promoting adaptive parenting.

## 1. Introduction

Establishing a definitive diagnosis of neurodevelopmental disorders in young children is challenging. In Japan, an average of 2 years and 1 month is required to confirm diagnoses [1]. Among neurodevelopmental disorders, autism spectrum disorder (ASD) may present with early signs, including delayed language development, marked fluctuations in behavioral responsiveness, and impairments in social communication, play, and motor development. However, establishing a definitive diagnosis before the age of 2 remains difficult [2]. Parents often notice a developmental delay in their children starting from 7 months to 3 years of ages (average: 1.5 years old); however, their children are not diagnosed with developmental disorders until 2–8 years of age (average: 4.0 years old) [3]. From the time parents detect developmental concerns to the confirmation of a diagnosis, they frequently experience substantial psychological stress [1]. Early responses to problematic behaviors, as well as other signs of developmental delay, are therefore essential to prevent exacerbation and secondary difficulties [4]. The diagnostic process for children with autism is a major source of stress for parents, who often express anxiety before seeking help from specialists and face delays or difficulties in establishing a diagnosis [5]. Parents of children with developmental disabilities report higher levels of mental health problems than physical health problems and higher anxiety scores than depression scores [6]. Regardless of diagnostic status, early intervention for children with behavioral difficulties and associated parenting challenges is critical to reduce the risk of the development of maladaptive parenting practices, protect parental mental health, and prevent potential abuse.

The Positive Parenting Program (Triple P) is an evidence-based parent training intervention designed to promote positive parenting practices and prevent disorders. It is a cognitive behavioral family intervention program designed for parents of children aged 2–12-years [7], and its effectiveness as a parenting program has been demonstrated for early-onset conduct problems in 3–12-year-olds [8]. Triple P supports parents in adopting constructive, consistent approaches to child rearing. The program comprises five levels of intervention: Level 1 provides universal, community-wide parenting information, whereas Level 5 provides intensive, individualized support for high-risk cases [7]. Evidence indicates that levels 2–5, which provide targeted guidance on core parenting strategies, are effective in many different settings, including Australia, the United States, Europe (e.g., the United Kingdom, Germany, and Switzerland), and East Asia (Japan, China, Hong Kong, and Singapore) [9].

Level 4 of Triple P is an intensive intervention that offers both group and individual approaches for parents struggling with serious problem behaviors or complex issues [10]. Level 4 is an effective early intervention for children at high risk of being diagnosed with neurodevelopmental disorders who have not been formally diagnosed [9]. Multiple meta-analyses have demonstrated its effectiveness in reducing behavioral problems among young children at risk for severe behavioral, emotional, and developmental disorders [11,12]. In Japan, mothers of children with behavioral issues identified during the age-3 health checkup, as well as mothers who find parenting difficult, are recruited to participate in Group Triple P (GTP), a Triple P Level 4 program [13]. GTP yielded benefits for both mothers and children, including reductions in child behavioral problems, improvements in parenting practices, decreases in maternal depression and parenting difficulties, and restoration of parenting confidence [9]. Similarly, Stepping Stones Triple P Level 4 for children with disabilities demonstrated positive outcomes among parents for children aged 2–14-year-olds [14]. At Level 4, GTP is designed to be non-stigmatizing and normalize parenting challenges, offering potential public health benefits as a population-based preventive approach [4]. For parents of children aged 3–6-year-olds, GTP improved their children’s behavioral problems, reduced dysfunctional parenting styles, and bolstered positive parenting practices, with effects sustained for at least 2 years [15].

Triple P yields benefits regarding children’s behaviors and the parenting of children ≤5.5 years of age [16]. For a mixed-age group of 3- and 4-year-olds, GTP decreased hyperactivity, attention deficits, and aggressive problems [17]. For a mixed-age group of 2- and 3-year-olds with physical aggression, GTP reduced aggression among the children and improved dysfunctional maternal parenting [18]. The high effectiveness of interventions for early-onset problem behaviors around 2–3 years of age highlights the need for preventive interventions at a young age [16]. While reviews have reported intervention studies involving children aged 0–18 years [9], the effectiveness of the Triple P intervention for 1–3-year-olds who have not received a definitive diagnosis of a neurodevelopmental disorder but exhibit behavioral, emotional, or developmental problems, or for families with children aged 3 or younger who experience parenting difficulties due to their children’s behavioral problems, has not yet been established. Children in this age range are often in the early stages of symptom emergence, and many parents experience confusion and psychological stress in response to their child’s behavioral difficulties. For example, a study on toddlers with autism symptoms found significant associations between child symptoms and parenting stress [19]. Delays or deficits in social relatedness were also associated with elevated overall parenting stress, parental distress, and parent–child relationship difficulties. In addition, evidence suggests that parenting has the greatest impact on attention-deficit/hyperactivity disorder (ADHD) in early childhood, with family effects diminishing as children age [20]. These findings underscore the need for timely support at both the family and community levels. Families awaiting diagnostic clarification often experience a service gap during which parental stress increases, while access to formal support remains limited. Scalable, group-based parenting programs delivered in community settings are expected to help bridge this gap and contribute to propose as a form of early intervention based on a population-based selective approach.

### Research Questions

To examine GTP as an early intervention for parents of younger children with suspected but undiagnosed neurodevelopmental disorders, we extracted data for 1–3-year-olds children who participated in a GTP program delivered to 60 mothers of 0–10-year-olds and analyzed changes before and after the intervention. for this subgroup. The purpose of this study was to evaluate the benefits of Triple P Level 4, GTP, as an early intervention for parents of 1–3-year-olds with potential neurodevelopmental disorders in community parenting support centers. Although this study is exploratory and informed by prior evidence [11,15], we hypothesized that GTP would reduce parental perceived child behavioral problems, decrease dysfunctional parenting practices, and improve maternal parenting adjustment. We therefore hypothesized that changes would be observed with a medium effect size (0.3 ≤ r < 0.5).

## 2. Materials and Methods

### 2.1. Study Design and Sampling

GTP was delivered as a parenting support program sponsored by Matsuyama City, Ehime Prefecture, to strengthen local maternal and child health services. To implement parenting support services in a real community-health setting, a non-randomized pre–post test design without a control group was adopted. Since recruitment only occurred once a year, we did not establish a control group or create a waiting list. Convenience sampling was employed. Participants were recruited through online public announcements, posters displayed at community parenting support centers, and referrals from childcare staff at daycare centers in Matsuyama City. Japan comprises 47 prefectures, each containing multiple municipalities. As of 2024, Matsuyama City, the capital of Ehime Prefecture, had a population of approximately 515,000 and a birth rate of 6.19 per 1000 individuals in the population. Given the exploratory nature of this study, we referred to reports indicating that a high effect size was observed in meta-analyses of studies with 35 or more participants [9].

### 2.2. Participants

**Figure 1:**
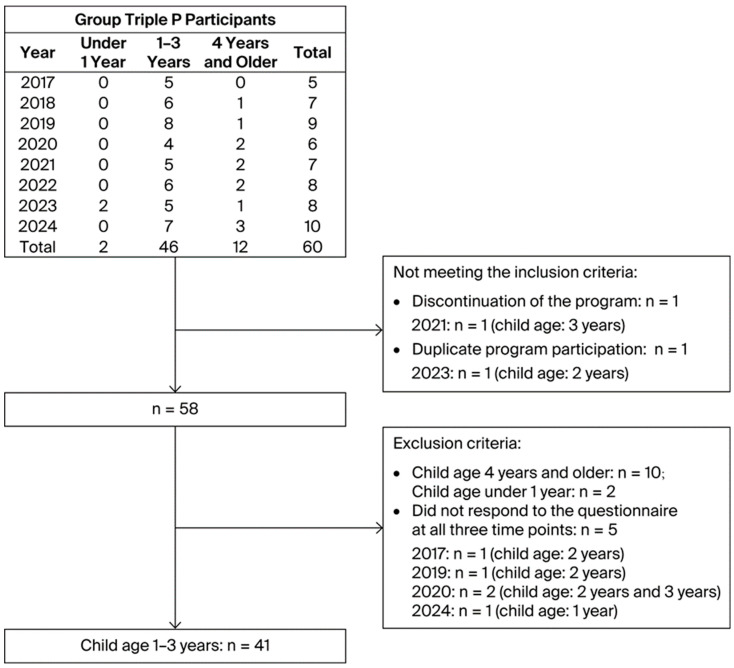
Participant selection process.

All participants were assigned to the intervention group. GTP was delivered at community parenting support centers in Matsuyama, Ehime Prefecture, from 2017 to 2024 (Figure 1). The participants were ① mothers of children for whom physicians or public-health nurses had identified developmental concerns during the 18-month health checkup or follow-up consultations or ② mothers who exhibited behavioral difficulties or parenting challenges according to observations made by public-health nurses or child-support-center staff. No screening tools were used. While the recruitment materials did not restrict participation by sex, only mothers enrolled. Mothers with more than one child were asked to select one target child for evaluation and to complete parenting assessments specific to that child.

Participants were included if they (1) attended at least two of the first four group sessions, (2) participated in the entire program, and (3) were first-time participants. One mother withdrew after the first session in 2021 for unknown reasons. Another mother participated in both the 2022 and 2023 programs; only her 2022 data were included in the analysis.

Participants were excluded if their children were under 1 year of age or older than 4 at program initiation. Children aged 12 months at baseline were classified as younger than 1 year, and those aged 47 months were classified as older than 4 years. Participants were also excluded if they had missing questionnaire data at any of the three assessment points (preintervention, postintervention, and 12 weeks follow-up).

### 2.3. Methods of Intervention

**Figure 2:**
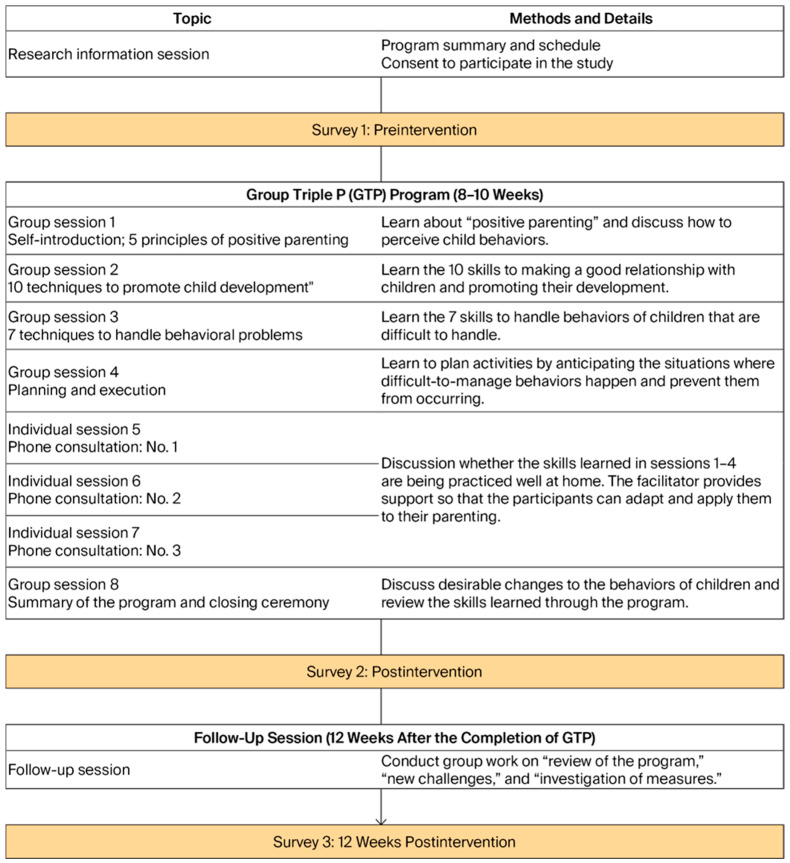
Methods of intervention.

GTP is a Triple P level 4 group-based intervention program, an evidence-based parenting intervention suitable for parents of children aged 2–12 who experience difficulties with parenting or whose children experience behavioral problems [11]. GTP comprises eight sessions conducted over approximately 8 weeks; of these, sessions 1–4 involved group learning for 5–10 participants for approximately 120 min, introducing positive parenting, training parents to record behaviors, and using role-playing for skill acquisition (Figure 2). Sessions 5–7 consisted of 15–20 min individual phone consultations, which evaluated parents’ implementation of parenting skills at home, and a facilitator provided suggestions for improvement. On the last day (session 8), the participants were asked to reconvene in their groups for a summary session and were guided to reflect on their learning to foster continued positive parenting at home. The participants also engaged in a follow-up session 12 weeks after completing GTP; they discussed how they practiced positive parenting at home and any new challenges they faced. Individual make-up sessions were provided for participants who were absent. The sessions were facilitated by eight Triple-P-certified facilitators (i.e., they had attended a 3-day course and passed the certification examination 6 months later, organized by Triple P Japan). The facilitator followed a standardized manual and a shared script. To ensure the program was of high quality, we recorded the participants’ remarks during group and individual sessions (with their consent) and prepared verbatim transcripts. After the group sessions, we conducted a debriefing among staff members. The participants were provided with free textbooks, childcare, and insurance coverage during the program.

### 2.4. Methods of Data Collection

The reliability and validity of the Japanese versions of the instruments used in this study have been confirmed in previous research. After the preliminary information session, the research outline was explained to the 60 participants, and their written informed consent was obtained. The questionnaire surveyed children’s sex, age, family composition, indications in health checkups, support from public health nurses and specialists, and childcare support. We investigated the effects of GTP on children’s behavior (Strengths and Difficulties Questionnaire, SDQ) [21], parenting style (Parenting Scale, PS) [22], perceptions of childcare (Parental Experience Survey, PES) [9], and parenting adjustment (Depression–Anxiety–Stress Scale, DASS) [23]. We employed a paper-based survey instrument at the following three intervals: pre-GTP (before the first session), post-GTP (after the eighth session), and 12 weeks after GTP completion.

The SDQ measures five factors that describe parents’ perceptions of their children’s socially desirable and undesirable behaviors—i.e., prosocial attitudes, peer problems, hyperactivity, conduct problems, and emotional problems—via 25-items rated on a three-point Likert scale (0–2) [13,14,21,24,25]. A modified version applicable to children aged 2 to 4 was adopted, and its application to 2-year-olds has been reported [26,27]. The total difficulty score is calculated as the sum of 20 items in four factors, excluding the 5 items of the prosocial scale (range, 0–40). The SDQ sets four evaluation segments (bandings) for 2- to 4-year-olds: close to average—approximately 80%; slightly raised/lowered—12%; high/low—4%; and very high/low—4%. SDQ was interpreted according to established cutoff ranges: for emotional problems, 0–2 = normal, 3 = borderline, 4 = slightly raised, and 5–10 = high; for conduct problems, 0–3 = normal, 4 = borderline, 5 = slightly raised, and 6–10 = high; for hyperactivity, 0–5 = normal, 6 = borderline, 7 = slightly raised, and 8–10 = high; for peer problems, 0–2 = normal, 3 = borderline, 4 = slightly raised, and 5–10 = high; for prosocial behavior, 7–10 = high, 6 = slightly lowered, 5 = low, and 0–4 = very low; and for total difficulties, 0–12 = normal, 13–15 = borderline, 16–18 = slightly raised, and 19–40 = high. Participants’ scores at each time point were classified according to these categories.

The PS evaluates dysfunctional parenting styles, including verbosity (lengthy verbal responses and reliance on talking even when talking is ineffective), over-reactivity (mistakes such as displays of anger, meanness, and irritability), and laxness (i.e., parents give in, allow rules to go unenforced, or provide positive consequences for misbehavior), through 30 items evaluated on a 7-point scale [22,28]. The internal consistency of the PS was tested using the reliability–retest method [13,14,25]. The means of the total score and subscales for mothers (*n* = 26) with children aged 18–48 months in the clinic group [22] were used as cutoff points to classify the participants into clinical and nonclinical groups. The clinical cutoff means were as follows: laxness (mean = 2.8; SD = 1.0), over-reactivity (mean = 3.0; SD = 1.0), verbosity (mean = 3.4; SD = 1.0), and total score (mean = 3.1; SD = 0.7). Scores above these means were classified under clinical range, and scores below the means were classified under normal range.

The PES evaluates six items taken from the Living With Children Survey [29,30]—(1) parenting is difficult, (2) experience of parenting (parenting is rewarding, demanding, stressful, fulfilling, or depressive), (3) confidence in parenting, (4) support for parenting, (5) agreement with one’s partner regarding child discipline, and (6) support received from one’s partner rated on a 5-point scale [9,13,14]—and one item from the dyadic adjustment scale [31], i.e., happiness with a partner, evaluated on a 7-point scale.

The DASS evaluates parental stress, anxiety, and depression using 42 items via a 4-point scale. The subscales are evaluated on five levels (normal to extremely severe), where moderate and higher levels are classified in the clinical range [13,14,23,25]. For depression, 0–9 = normal; 10–13 = mild; 14–20 = moderate; 21–27 = severe; and 28+ = extremely severe. For anxiety, 0–7 = normal; 8–9 = mild; 10–14 = moderate; 15–19 = severe; and 20+ = extremely severe. For stress, 0–14 = normal; 15–18 = mild; 19–25 = moderate; 26–33 = severe; and 34+ = extremely severe. Scores in this study were categorized according to these severity levels.

### 2.5. Statistical Analysis

Prior to analysis, the dataset was examined to confirm the absence of missing values. Given the small sample size and the fact that the Kolmogorov–Smirnov normality test and the Shapiro–Wilk test indicated that the variable distributions were not normal, nonparametric tests were applied (K–S: *D* = 0.161–0.312, *p* < 0.05; S–W: *W* = 0.623–0.986, *p* < 0.05). Details are provided in the Appendix A. Friedman’s test was used to compare outcomes across the three assessment points (preintervention, postintervention, and the 12-week follow-up), with statistical significance set at *p* < 0.05 [32]. When significant differences were detected, post hoc pairwise comparisons were conducted using the Wilcoxon signed-rank test with Bonferroni adjustment (*p* < 0.017). Given that small sample sizes may limit statistical power, effect sizes were calculated to assess practical significance using *r* = Z/√N [33]. Effect sizes were interpreted as small (0.1 ≤ *r* <0.3), medium (0.3 ≤ *r* < 0.5), or large (*r* ≥ 0.5) [34,35]. We reported the Hodges–Lehmann (HL) estimate and its 95% confidence interval as a measure of the effect of the median difference. All analyses were conducted using Microsoft Excel 2019 (Microsoft Corp., Redmond, WA, USA) and IBM SPSS Statistics (Version 26; IBM Corp., Armonk, NY, USA).

## 3. Results

A total of 41 mothers participated in this study (Table 1). Recruitment sources included parent–child classes (*n* = 19), parenting support centers (*n* = 7), self-referrals from mothers (*n* = 4), and other sources (*n* = 11). Attendance rates were 85.6% for Sessions 1–4 and 91.5% for the full program. The mean age of the target children was 2.1 years (30.7 months; SD = 0.7 years/7.6 months). Most of the children were firstborns (*n* = 35, 85.4%).

GTP promoted the mothers’ ability to evaluate positive behaviors in their 1–3-year-olds with potential neurodevelopmental disorders. The SDQ: the prosocial scales were significantly higher after the intervention and at 12 weeks postintervention compared to preintervention, indicating a medium effect size (*r* = 0.372, 0.373, 95% CI [0.500, 2.000], [0.500, 1.500], *p* < 0.001) (Table 2 and Table 3). The evaluation category was “very low” at preintervention but improved to “low” both after the intervention and at 12 weeks postintervention (Table 4). Factors related to challenging behaviors: emotional problems, conduct problems, hyperactivity, peer problems were no significant difference. The evaluation category for peer problems remained at “slightly raised”, while other factors remained at a level “close to average.”

GTP improved overly reactive parenting styles for mothers of children aged 1–3 years. PS over-reactivity was significantly lower postintervention than preintervention, decreasing with large and medium effect sizes (*r* = 0.557, 0.440, 95% CI [−1.100, −0.600], [−0.900, −0.350], *p* < 0.001). Over-reactivity was in the clinical range preintervention and within the normal range postintervention and at 12 weeks postintervention. The total score was significantly lower postintervention and 12 weeks postintervention than it was preintervention, decreasing with large effect sizes (*r* = 0.548, 0.504, 95% CI [−0.667, −0.383], [−0.500, −0.233], *p* < 0.001). Laxness was significantly lower postintervention and at 12 weeks postintervention than preintervention, decreasing with medium effect sizes (*r* = 0.393, 0.319, 95% CI [−0.636, −0.227], [−0.500, −0.091], *p* < 0.001, *p* < 0.017). Verbosity was significantly lower postintervention than preintervention; it decreased with small effect sizes postintervention (*r* = 0.297, 95% CI [−0.500, −0.1431], *p* < 0.017). The evaluation categories for laxness, verbosity, and total score remained unchanged from the clinical domain.

With respect to perceptions of parenting, GTP restored parenting confidence and sense of fulfillment in mothers, and reduced parent depression. The PES item, “Confidence in parenting” was significantly higher postintervention and at 12 weeks postintervention than preintervention, increasing with a medium effect size (*r* = 0.377, 0.440, 95% CI [0.500, 1.000], [0.500, 1.000], *p* < 0.001). “Parenting is fulfilling” was significantly higher postintervention than preintervention, increasing with a medium effect size (*r* = 0.413, 95% CI [0.500, 1.500], *p* < 0.001). “Parenting is depressive” (a reversal item), was significantly higher postintervention than preintervention, increasing with a medium effect size (*r* = 0.363, 95% CI [0.500, 1.000], *p* < 0.017).

GTP also improved their perceptions of receiving parenting support. “Support for parenting” was significantly higher postintervention than preintervention, increasing with a medium effect size (*r* = 0.331, 95% CI [0.500, 1.000], *p* < 0.017).

Maternal mental health remained within the normal range, but it declined over the course of the 12-week follow-up. Stress and total scores on the DASS were significantly lower at 12 weeks than they were preintervention, decreasing with a medium effect size (*r* = 0.370, 0.370, 95% CI [−7.000, −1.500], [−14.000, −2.500], *p* < 0.001). Anxiety and depression were also significantly lower at 12 weeks than they were preintervention, decreasing with a small effect size (*r* = 0.300,0.278, 95% CI [−2.500, −0.500], [−4.000, −0.500], *p* < 0.017). The DASS total score and the three subscales were within the normal range at all three measurement points.

For some items, statistically significant differences were observed, and small effect sizes were noted; however, reliability could not be confirmed, leaving the results uncertain. For example, “Parenting is difficult” (preintervention to postintervention, *r* = 0.276, 95% CI [0.000, 0.500], *p* < 0.017) and “Agreement with partner regarding child discipline” (preintervention to 12 weeks postintervention, *r* = 0.373, 95% CI [0.000, 0.500], *p* < 0.017).

## 4. Discussion

This study evaluated the benefits of Group Triple P (GTP) among mothers of children aged 1 to 3 years who may have neurodevelopmental disorders; its novelty lies in the finding that mothers’ assessments of their children’s positive behaviors, as measured by the same scale (SDQ), improved. The increase in the prosocial score was statistically significant, and the effect size was medium. The evaluation category improved from “very low” before the intervention to “low” after the intervention and remained at that level through 12 weeks. On the other hand, no significant changes were observed in the perception of children’s problem behaviors; peer problems remained “slightly raised”, while other factors remained within the “close to average” range. These changes are based on mothers’ subjective evaluations and do not directly indicate objective changes in children’s behaviors or abilities. Meta-analyses have reported that parental assessments can yield results comparable to those of observers [36]. A meta-analysis of 55 studies across Triple P Levels 1–5 reported mixed findings on whether the program increases positive behaviors or reduces negative behaviors in children [16]. The ages of 1 to 3 years are a period of marked developmental change; to distinguish between variations due to natural history (maturation) and intervention effects, an evaluative design with a control group is essential. This may be one of the reasons why reliability could not be ensured in some of the tests. Moving forward, it is necessary to verify the reproducibility of mother-reported findings through the introduction of RCTs with sufficient sample sizes and control groups, as well as observer ratings, and to empirically evaluate the relationship between changes in parenting behaviors and changes in child outcomes through mediation analyses.

Previous studies involving children aged 3 years and older have reported a reduction in problem behaviors rather than changes in positive behaviors. In Japan, GTP significantly reduced conduct problems on the SDQ for children with a mean age of 3.1 years (SD = 1.35) [13]. In Hong Kong, GTP, when delivered to children with a mean age of 4.23 years (SD = 1.06) significantly improved peer problems, hyperactivity, conduct problems, and emotional problems on the SDQ [37]. While this study is largely comparable to previous research in terms of cultural context (Asia), measurement scale (SDQ), and intervention framework (GTP), its primary limitations lie in the small sample size and the study design’s lack of a control group. Therefore, comparisons are limited to descriptive references based on the continuity of the framework, and we do not intend to generalize the benefits. However, differences may exist between interventions for children aged 1–3 and those for children aged 3 and older, suggesting that there is need to further examine the significance of early intervention.

GTP had a positive benefit in improving maternal dysfunctional parenting styles. Over-reactivity was consistent with a shift from the clinical range to the normal range, and the effect size was large. In contrast, for laxness and verbosity, the effect sizes were medium and small, and the evaluation remained in the clinical range. While these results suggest some improvement in terms of reducing over-reactivity, it is necessary to determine whether changes in daily interaction patterns—such as consistency in discipline and the frequency of verbal interventions—depend on the child’s age or on parental characteristics. Previous studies have also reported improvements in parenting styles, including each subscale of the PS [13,25,37]. Although studies in East Asia have found that the majority of participants are mothers and have confirmed the cultural acceptability of GTP, caution is warranted in this study regarding interpretive limitations arising from gender bias. Negative parenting practices are associated with increased emotional and behavioral problems in children, whereas modifying maladaptive parenting styles contributes to the primary and secondary prevention of behavioral difficulties [28,38]. However, this study did not directly examine the causal mechanisms or preventive effects related to the improvement of children’s behavioral problems; furthermore, due to limitations in the study design, caution is required when interpreting the extent to which GTP contributes to mothers’ selection of appropriate parenting behaviors. To further investigate this point, it would be useful to incorporate qualitative research. The background behind laxness and verbosity reaching clinical levels in children aged 1–3 years, as well as the process by which they subside after age 3, are beyond the scope of this study; therefore, it is necessary to evaluate how changes in parenting styles within the target age group progress and become established over time.

GTP also brought about positive changes in the mothers’ psychological outcomes. In this study, DASS scores remained within the normal range throughout the study; the total score decreased from 21.85 (SD = 23.19) before the intervention to 12.29 (SD = 14.70) after 12 weeks, showing a medium effect size. Implementing GTP when parents’ mental health is within the normal range may contribute to maintaining mental well-being and preventing deterioration, and can be positioned as a “population-based selective approach” targeting risk-based subgroups. For reference, parents of children with neurodevelopmental disorders, with a mean age of 7.4 years (SD = 2.7), reported a mean DASS total score of 26.22 (SD = 18.33) [14], and parents of children with autism in Asia have been shown to exhibit significantly higher levels of depression and anxiety [39]. Given that neglecting the burden of caregiving can lead to an increase in mothers’ psychological stress, the decrease in DASS scores observed in this study is considered a change consistent with the maintenance of well-being and the prevention of deterioration. Nevertheless, conclusions regarding clinical significance cannot be drawn within the scope of this study. As GTP is a group-based program, it is suggested that interaction among mothers facing similar challenges may provide emotional support; however, because this study did not directly measure the specific processes of peer support, there are limitations to interpreting this finding. While this study suggested improvements in perceptions regarding access to parenting support, causal relationships cannot be identified from this study. It is necessary to qualitatively analyze and verify the mothers’ experience.

The benefits of GTP were observed up to 12 weeks postintervention, including an increase in mothers’ recognition of their children’s positive behaviors, a restored parenting style and confidence in parenting, and sustained mental well-being. A 2-year follow-up study in another context found sustained reductions in dysfunctional parenting and continued improvements in child behavior [15]. In contrast, a meta-analysis reported that improvements in dysfunctional maternal parenting were not consistently maintained at the 6-month follow-up [12]. Given that this study included only a 12-week follow-up, future research should incorporate longer-term assessments beyond 6–12 months to determine the durability of the intervention’s effects.

### 4.1. Study Strengths

This study is novel in that it evaluated the benefits of GTP (Group Triple P) among mothers of children aged 1–3 years who may have neurodevelopmental disorders, a population for which there is limited prior research. We utilized validated psychometric instruments, and implementation within a real-world community health setting enhanced this study’s ecological validity.

### 4.2. Limitations

Several limitations warrant consideration. First, because this was an open-label intervention without a control group, the ability to make causal inferences regarding the efficacy of GTP is limited. The improvements observed may reflect developmental maturation, regression to the mean, or increased parental awareness associated with repeated measurements. Given that the participants self-enrolled, a bias due to increased awareness cannot be ruled out. Second, statistical power was limited by the relatively small sample size, and generalizability was restricted because the participants consisted solely of mothers from a single municipality in Japan, where nuclear families are prevalent. This study was based on actual community-based child-rearing support practices and did not include exclusion criteria such as maternal mental illness. Third, outcomes were based solely on mothers’ subjective assessments, and positive child behaviors were assessed using a single subscale of the SDQ, indicating a need for more comprehensive, developmentally appropriate measures. Finally, only short-term outcomes (12 weeks) were evaluated; thus, the durability of the intervention’s effects remains uncertain.

### 4.3. Future Directions

Further RCTs must be paired with long-term follow-up studies conducted using objective measures. Meta-analyses of confounding factors—such as participant gender—using large samples are also necessary to generate stronger evidence regarding the efficacy of GTP.

## 5. Conclusions

GTP was found to benefit mothers of children who may have neurodevelopmental disorders in the pre-diagnostic stage as an early intervention, contributing to increased parent-reported recognition of children’s positive behaviors, improvements in parenting style, and the maintenance of maternal mental health. The intervention in this study targeted a subgroup at higher-than-average risk—specifically, mothers of children aged 1–3 years at risk for neurodevelopmental disorders—and therefore aligns with the concept of selective prevention, i.e., preventive intervention delivered before symptoms become manifest. Furthermore, implementing scalable, community-based parenting programs could help mitigate service disparities experienced by families awaiting a definitive diagnosis. From a public health perspective, the present findings suggest that GTP can be positioned as an adaptive, population-based selective approach for families of children who may have neurodevelopmental disorders prior to formal diagnosis. However, this study’s modest sample size limits its generalizability. Future research should accumulate longitudinal evidence gathered using reliable, developmentally appropriate measures to evaluate the sustainability of the intervention’s effects.

## Figures and Tables

**Table 1:** Overview of the participants.

Variable	Item	*n* (%)	Mean (SD)	Range
Recruitment route	Referred from the parenting class	19 (46.3)	-	-
	Referred from the childcare support center	7 (17.1)	-	-
	Direct recruitment of voluntary mothers	4 (9.8)	-	-
	Other	11 (26.8)	-	-
Attendance rates	Sessions 1–4	85.6	-	-
	Total program	91.5	-	-
Participants	Sex (woman)	41 (100)	-	-
	Age (years)	-	35.73 (4.29)	25–43
Spouse	In spousal relationship with the subject	41 (100)	-	-
	Age (years)	40	37.42 (6.29)	27–52
Family type	Nuclear family	39 (95.1)	-	-
	Extended family	2 (4.9)	-	-
Child	Sex (boy)	31 (75.6)	-	-
	Sex (girl)	10 (24.4)	-	-
	Age (years)	-	2.10 (0.70)	1–3
	Age (months)	-	30.71 (7.60)	18–45
	Firstborn	25 (85.4)	-	-
	History of attending 1-year–6-month health checkups	41 (100)	-	-
Public support	Registration with the parenting class at the childcare support center	19 (46.3)	-	-
	Receiving care	4 (1.1)	-	-

**Table 2:** Evaluation of the mothers.

Scale	Factor/Subscale Subcategory	Pre Mean (SD)	Post Mean (SD)	12 Weeks Mean (SD)	Friedman *p*	Post Hoc *p* (Pre–Post/Post-12 Weeks/Pre-12 Weeks)	*r* (Pre–Post/Post-12 Weeks/Pre-12 Weeks)
SDQ	Emotional problems scale	2.44 (1.98)	2.49 (2.16)	2.00 (2.09)	0.156	0.709/0.085/0.159	0.041/0.190/0.156
	Conduct problems scale	2.73 (1.72)	2.56 (1.48)	2.39 (1.53)	0.627	0.411/0.413/0.192	0.091/0.090/0.144
	Hyperactivity scale	3.71 (1.54)	3.51 (1.79)	3.44 (1.50)	0.805	0.580/0.803/0.226	0.061/0.027/0.134
	Peer problems scale	3.10 (1.41)	3.17 (1.45)	3.34 (1.32)	0.584	0.687/0.383/0.328	0.045/0.096/0.108
	Prosocial scale ^a^	4.27 (2.41)	5.49 (2.37)	5.39 (2.23)	***	***/0.571/***	0.372/0.063/0.373
	Total difficulties score	11.98 (4.26)	11.73 (4.31)	11.17 (3.77)	0.682	0.770/0.170/0.273	0.032/0.152/0.121
PS	Laxness	4.04 (0.61)	3.61 (0.65)	3.74 (0.67)	*	***/0.062/**	0.393/0.206/0.319
	Over-reactivity	3.40 (1.28)	2.52 (0.95)	2.74 (0.92)	***	***/0.027/***	0.557/0.245/0.440
	Verbosity	3.79 (0.63)	3.52 (0.63)	3.59 (0.70)	*	**/0.348/0.042	0.297/0.104/0.225
	Total score	3.71 (0.55)	3.20 (0.49)	3.33 (0.51)	***	***/0.022/***	0.548/0.253/0.504
PES ^a^	(1) Parenting is difficult ^b^	3.07 (0.98)	3.44 (0.74)	3.49 (0.68)	*	**/0.723/0.033	0.276/0.039/0.236
	(2) Experience of parenting:						
	① Parenting is rewarding	3.20 (1.05)	3.56 (1.23)	3.39 (1.07)	0.137	0.075/0.323/0.340	0.197/0.109/0.105
	② Parenting is demanding ^b^	2.88 (1.05)	3.32 (0.96)	3.34 (0.96)	0.051	0.019/0.855/**	0.260/0.020/0.286
	③ Parenting is stressful ^b^	3.20 (1.10)	3.39 (1.00)	3.51 (0.93)	0.134	0.310/0.568/0.067	0.112/0.063/0.202
	④ Parenting is fulfilling	2.71 (1.05)	3.66 (1.22)	3.07 (1.15)	***	***/**/0.054	0.413/0.358/0.213
	⑤ Parenting is depressive ^b^	3.17 (1.12)	3.85 (0.96)	3.85 (0.91)	*	**/0.891/**	0.363/0.015/0.332
	(3) Confidence in parenting	2.24 (0.92)	2.83 (0.80)	2.88 (0.78)	***	***/0.686/***	0.377/0.045/0.440
	(4) Support for parenting	2.95 (0.97)	3.68 (0.93)	3.46 (1.03)	*	**/0.242/**	0.331/0.129/0.299
	(5) Agreement with partner regarding child discipline	3.00 (0.95)	3.44 (1.05)	3.44 (0.87)	*	**/0.870/***	0.265/0.018/0.373
	(6) Support received from partner	3.29 (1.21)	3.61 (1.22)	3.56 (1.21)	*	0.042/0.658/0.033	0.225/0.049/0.236
	(7) Happiness with partner	3.49 (1.25)	3.73 (1.28)	3.80 (1.14)	0.063	0.049/0.499/0.042	0.217/0.075/0.225
DASS	Depression	6.20 (7.50)	4.66 (7.94)	3.61 (5.41)	*	0.040/0.319/**	0.227/0.110/0.278
	Anxiety	3.98 (6.02)	2.07 (3.55)	1.83 (3.19)	*	0.018/0.425/**	0.261/0.088/0.300
	Stress	11.68 (10.75)	8.51 (8.16)	6.85 (6.94)	*	0.031/0.070/***	0.238/0.200/0.370
	Total score	21.85 (23.19)	15.24 (18.22)	12.29 (14.70)	*	0.025/0.093/***	0.248/0.186/0.370

Note. ^a^: A high score indicates a good result. ^b^: Reverse-scored items, * *p* < 0.05, ** *p* < 0.017, *** *p* < 0.001. Pre: preintervention; Post: postintervention; 12 weeks: 12 weeks postintervention.

**Table 3:** Post hoc statistical measures.

Scale	Factor/Subscale Subcategory	Z (Pre–Post/Post-12 Weeks/Pre-12 Weeks)	95% CI (Pre–Post/Post-12 Weeks/Pre-12 Weeks)
SDQ	Emotional problems scale	−0.373/−1.725/−1.409	[−0.500, 0.000]/[0.000, 1.000]/[−1.000, 0.000]
	Conduct problems scale	−0.823/−0.819/−1.304	[−0.500, 0.000]/[0.000, 0.500]/[−0.500, 0.000]
	Hyperactivity scale	−0.554/−0.249/−1.211	[−1.000, 0.500]/[−0.500, 0.500]/[−0.500, 0.500]
	Peer problems scale	−0.403/−0.873/−0.979	[−0.500, 0.500]/[−0.500, 0.000]/[0.000, 0.500]
	Prosocial scale ^a^	−3.370/−0.567/−3.380	[0.500, 2.000]/[−0.500, 1.000]/[0.500, 1.500]
	Total difficulties score	−0.292/−1.373/−1.096	[−1.000, 1.000]/[−0.500, 1.500]/[−0.500, 2.000]
PS	Laxness	−3.562/−1.869/−2.886	[−0.636, −0.227]/[−0.318, 0.000]/[−0.500, −0.091]
	Over-reactivity	−5.044/−2.217/−3.986	[−1.100, −0.600]/[−0.400, 0.000]/[−0.900, −0.350]
	Verbosity	−2.693/−0.938/−2.033	[−0.500, −0.143]/[−0.357, 0.143]/[−0.429, 0.000]
	Total score	−4.964/−2.291/−4.565	[−0.667, −0.383]/[−0.233, −0.017]/[−0.500, −0.233]
PES ^a^	(1) Parenting is difficult ^b^	−2.499/−0.354/−2.138	[0.000, 0.500]/[0.000, 0.000]/[0.000, 1.000]
	(2) Experience of parenting:		
	① Parenting is rewarding	−1.780/−0.988/−0.955	[0.000, 0.500]/[−0.500, 0.000]/[0.000, 0.500]
	② Parenting is demanding ^b^	−2.352/−0.183/−2.587	[0.000, 1.000]/[−0.500, 0.500]/[0.000, 0.500]
	③ Parenting is stressful ^b^	−1.014/−0.571/−1.830	[0.000, 0.500]/[−0.500, 0.000]/[0.000, 0.500]
	④ Parenting is fulfilling	−3.740/−3.245/−1.929	[0.500, 1.500]/[0.000, 1.000]/[0.000, 1.000]
	⑤ Parenting is depressive ^b^	−3.285/−0.138/−0.003	[0.500, 1.000]/[−0.500, 0.500]/[0.000, 1.000]
	(3) Confidence in parenting	−3.417/−0.404/−3.984	[0.500, 1.000]/[−0.500, 0.000]/[0.500, 1.000]
	(4) Support for parenting	−2.995/−1.170/−2.706	[0.500, 1.000]/[0.000, 0.500]/[0.000, 1.000]
	(5) Agreement with partner regarding child discipline	−2.400/−0.164/−3.382	[0.000, 0.500]/[0.000, 0.000]/[0.000, 0.500]
	(6) Support received from partner	−2.039/−0.443/−2.138	[0.000, 0.500]/[0.000, 0.000]/[0.000, 0.500]
	(7) Happiness with partner	−1.966/−0.676/−2.034	[0.000, 0.500]/[0.000, 0.000]/[0.000, 0.500]
DASS	Depression	−2.057/−0.996/−2.521	[−3.500, 0.000]/[−0.500, 1.000]/[−4.000, −0.500]
	Anxiety	−2.361/−0.798/−2.716	[−2.000, 0.000]/[0.000, 0.500]/[−2.500, −0.500]
	Stress	−2.151/−1.809/−3.347	[−4.000, 0.000]/[0.000, 3.500]/[−7.000, −1.500]
	Total score	−2.249/−1.681/−3.351	[−9.500, −0.500]/[−0.500, 5.000]/[−14.000, −2.500]

Note. ^a^: A high score indicates a good result. ^b^: Reverse-scored items. Pre: preintervention; Post: postintervention; 12 weeks: 12 weeks postintervention.

**Table 4:** Changes to the indicators preintervention, postintervention, and 12 weeks postintervention.

Scale	Factor/Subscale Subcategory	Pre-Mean/Evaluation	Post-Mean/Evaluation	12-Weeks Mean/Evaluation	Cronbach’s α
SDQ	Emotional problems scale	2.44/Close to average	2.49/Close to average	2.00/Close to average	0.83
	Conduct problems scale	2.73/Close to average	2.56/Close to average	2.39/Close to average	0.84
	Hyperactivity scale	3.71/Close to average	3.51/Close to average	3.44/Close to average	0.76
	Peer problems scale	3.10/Slightly raised	3.17/Slightly raised	3.34/Slightly raised	0.75
	Prosocial scale ^a^	4.27/Very low	5.49/Low	5.39/Low	0.84
	Total difficulties score	11.98/Close to average	11.73/Close to average	11.17/Close to average	0.84
PS	Laxness	4.04/Clinical range	3.61/Clinical range	3.74/Clinical range	0.80
	Over-reactivity	3.40/Clinical range	2.52/Normal range	2.74/Normal range	0.90
	Verbosity	3.79/Clinical range	3.52/Clinical range	3.59/Clinical range	0.55
	Total score	3.71/Clinical range	3.20/Clinical range	3.33/Clinical range	0.87
DASS	Depression	6.20/Normal	4.66/Normal	3.61/Normal	0.81
	Anxiety	3.98/Normal	2.07/Normal	1.83/Normal	0.82
	Stress	11.68/Normal	8.51/Normal	6.85/Normal	0.83

Note. ^a^: A high score indicates a good result. Pre: preintervention; Post: postintervention; 12 weeks: 12 weeks postintervention.

## Data Availability

The datasets generated and/or analyzed during this study are available from the corresponding author on reasonable request.

## Abbreviations

The following abbreviations are used in this manuscript:

Triple P	Positive Parenting Program
GTP	Group Triple P
ADHD	Attention-Deficit/Hyperactivity Disorder
ASD	Autism Spectrum Disorder
SDQ	Strengths and Difficulties Questionnaire
PS	Parenting Scale
PES	Parental Experience Survey
DASS	Depression–Anxiety–Stress Scale

## Appendix A. Test for Normality

**Table:**

Scale	Factor/ Subscale Subcategory	Kolmogorov–Smirnov Test *D·p*						Shapiro–Wilk Test *W·p*					
		Pre		Post		12-Weeks		Pre		Post		12-Weeks	
SDQ	Emotional problems scale	0.181	**	0.169	**	0.173	**	0.914	**	0.880	**	0.850	**
	Conduct problems scale	0.250	**	0.262	**	0.259	**	0.857	**	0.764	**	0.805	**
	Hyperactivity scale	0.229	**	0.144	*	0.184	**	0.930	*	0.953	0.089	0.950	0.068
	Peer problems scale	0.162	**	0.181	**	0.212	**	0.950	0.069	0.943	*	0.931	*
	Prosocial scale ^a^	0.163	**	0.122	0.128	0.114	**	0.950	0.067	0.964	0.213	0.970	0.356
	Total difficulties score	0.161	**	0.109	**	0.117	0.177	0.940	*	0.963	0.206	0.940	*
PS	Laxness	0.073	**	0.055	**	0.105	**	0.979	0.636	0.983	0.788	0.975	0.478
	Over-reactivity	0.115	**	0.131	0.074	0.078	**	0.949	0.063	0.913	**	0.983	0.791
	Verbosity	0.125	**	0.126	0.102	0.100	**	0.944	*	0.964	0.222	0.963	0.198
	Total score	0.074	**	0.133	0.067	0.108	**	0.986	0.894	0.967	0.276	0.960	0.156
PES	(1) Parenting is difficult	0.241	**	0.311	**	0.312	**	0.870	**	0.806	**	0.798	**
	(2) Experience of parenting:												
	① Parenting is rewarding	0.208	**	0.197	**	0.204	**	0.914	**	0.868	**	0.866	**
	② Parenting is demanding	0.198	**	0.298	**	0.224	**	0.892	**	0.846	**	0.903	**
	③ Parenting is stressful	0.231	**	0.242	**	0.262	**	0.888	**	0.889	**	0.868	**
	④ Parenting is fulfilling	0.212	**	0.220	**	0.215	**	0.897	**	0.857	**	0.861	**
	⑤ Parenting is depressive	0.308	**	0.292	**	0.222	**	0.834	**	0.846	**	0.867	**
	(3) Confidence in parenting	0.210	**	0.267	**	0.269	**	0.869	**	0.858	**	0.860	**
	(4) Support for parenting	0.187	**	0.291	**	0.285	**	0.907	**	0.851	**	0.872	**
	(5) Agreement with partner regarding child discipline	0.196	**	0.264	**	0.302	**	0.891	**	0.885	**	0.840	**
	(6) Support received from partner	0.223	**	0.189	**	0.179	**	0.864	**	0.866	**	0.876	**
	(7) Happiness with partner	0.189	**	0.204	**	0.226	**	0.924	**	0.895	**	0.925	**
DASS	Depression	0.204	**	0.279	**	0.252	**	0.779	**	0.623	**	0.712	**
	Anxiety	0.275	**	0.302	**	0.283	**	0.675	**	0.647	**	0.622	**
	Stress	0.172	**	0.184	**	0.196	**	0.859	**	0.865	**	0.837	**
	Total score	0.193	**	0.201	**	0.201	**	0.826	**	0.771	**	0.779	**

Note. ^a^, A high score indicates a good result. * *p* < 0.05, ** *p* < 0.01. Pre: preintervention; Post: postintervention; 12 weeks: 12 weeks postintervention.
